# Beyond inhibition: harnessing the DRD2–VEGF-A feedback loop for precision anti-angiogenesis therapy in cancer

**DOI:** 10.3389/fonc.2026.1818655

**Published:** 2026-05-28

**Authors:** Manas Ranjan Sahu, Venu Akkanapally, Partha Sarathi Dasgupta, Sujit Basu

**Affiliations:** 1 Department of Pathology, Ohio State University, Columbus, OH, United States; 2 Chittaranjan National Cancer Institute, Kolkata, India; 3 Department of Internal Medicine, Division of Medical Oncology, Columbus, OH, United States; 4 Comprehensive Cancer Center, Ohio State University, Columbus, OH, United States

**Keywords:** angiogenesis, cancer, dopamine D2 receptor agonists, therapy, vascular endothelial growth factor receptor-2, vascular endothelial growth factor-A

## Abstract

Current anti-angiogenic therapies using vascular endothelial growth factor-A/vascular endothelial growth factor receptor-2 (VEGF-A/VEGFR2) inhibitors lack dynamic biomarkers that can facilitate patient selection and optimize dosing; this can lead to suboptimal and unsatisfactory outcomes and significant toxicities. To address this, we propose a paradigm shift leveraging the dopamine D2 receptor (DRD2)/VEGF-A paracrine feedback loop. In the tumor microenvironment, VEGF-A selectively induces endothelial DRD2 expression through specific transcriptional mechanisms – a molecular signature that is absent in quiescent normal vasculature. DRD2 activation serves as a potent, tumor-specific vascular brake on VEGFR2 phosphorylation and paracellular permeability. By utilizing FDA-approved DRD2 agonists as functional probes, clinicians can implement a dopaminergic challenge to identify windows of maximal VEGF-dependency via dynamic imaging. This approach transforms anti-angiogenic interventions from empirical into a precision theranostic platform, enabling real-time assessment of tumor VEGF-dependency and rational treatment selection that maximizes efficacy while minimizing toxicity.

## Introduction

Although the era of anti-vascular endothelial growth factor (VEGF) therapy is two decades old, its clinical practice continues to rely on empirical dosing (1, 2) despite VEGF-A dependency fluctuating as tumors evolve or activate alternative escape pathways (3, 4). There remains a lack of a functional probe that can identify these transient windows of tumor vulnerability (2, 5). Consequently, patients often receive prolonged, nonselective blockading agents that lead to the anti-angiogenic paradox: excessive vascular pruning that induces hypoxia, promotes therapy resistance, and incurs systemic toxicities such as hypertension and thromboembolism (6, 7).

### A biologically tuned feedback system

Emerging evidence suggests that the tumor microenvironment harbors intrinsic regulatory mechanisms that are amenable to therapeutic exploitation. Among these, the dopamine D2 receptor (DRD2)–VEGF-A axis functions as a biological rheostat of angiogenesis (8). Consistent with this phenomenon, clinical studies of patients with giant pituitary tumors, a non-malignant tumor, have revealed that cabergoline, a potent ergot-derived DRD2 agonist, can suppress tumor growth through the inhibition of pathological angiogenesis and subsequent cellular proliferation (9). Unlike tyrosine kinase inhibitors, which indiscriminately attenuate systemic signaling (10), cabergoline and other DRD2 agonists such as quinagolide exert context-dependent inhibition (11–13). This mechanism preserves homeostatic vascular integrity while selectively targeting the aberrant tumor endothelium, thereby offering a superior therapeutic index as well as a promising avenue for precision modulation of the tumor microenvironment.

Mechanistically, VEGF-A activates the transcription factor KLF11 via the ERK1/2 pathway, which, in turn, upregulates DRD2 specifically in tumor endothelial cells. This establishes a self-regulating feedback loop where high VEGF-A levels prime the endothelium for DRD2-mediated suppression. Upon activation, DRD2 inhibits VEGF-A-induced phosphorylation of the tight junction protein ZO-1 as well as vascular endothelial-cadherin/catenin complexes at the adherens junction, thus preserving endothelial adhesion and restricting paracellular permeability (14). Additionally, DRD2 signaling promotes vascular maturation by upregulating endothelial KLF2 and stimulating pericyte-derived Ang-1 expression, thereby facilitating mural cell recruitment and vessel normalization to stabilize the endothelium against VEGF-A-driven remodeling (15).

Quiescent normal vessels express minimal or no DRD2 in the absence of sustained VEGF-A stimulation (8). This intrinsic biological phenomenon confers tumor-endothelial selectivity not achieved by nonselective anti-VEGF-A/VEGFR2 antibodies or tyrosine kinase inhibitors that can induce deleterious vascular pruning, intratumoral hypoxia, and systemic toxicity. Consequently, the VEGF-A–ERK1/2–KLF11 signaling axis emerges as a robust endothelial biomarker for predicting DRD2-agonist responsiveness; however, this insight warrants high-resolution spatial transcriptomic mapping to enable precision stratification across diverse tumor microenvironments.

### Lessons from vascular homeostasis

While classically recognized as a central neurotransmitter synthesized in the specialized neurons of the brain, dopamine is also substantially produced in mesenteric organs where it exerts pivotal peripheral effects (16). Beyond its established role in motor control, dopamine functions as a systemic homeostatic regulator that modulates cardiovascular, renal, and endocrine functions through the dynamic interplay of D1- and D2-like receptor signaling (17). Emerging preclinical evidence suggests that endothelial DRD2 activation suppresses VEGF-A-induced angiogenesis and vascular permeability in the tumor microenvironment by inhibiting VEGFR-2 phosphorylation and downstream MAPK/focal adhesion kinase signaling. Given the well-characterized safety profile of FDA-approved DRD2 agonists (e.g., cabergoline) (11–13, 18), this signaling axis positions endothelial DRD2 as a highly tractable therapeutic target for selectively attenuating VEGF-A-driven vascular dysfunction.

In non-oncologic clinical conditions involving pathological VEGF-A-driven vascular leak, such as ovarian hyperstimulation syndrome (OHSS), neovascular endometriosis, and sepsis-related lung injury, DRD2 agonists or dopamine acting through D2 receptors effectively mitigate edema and ascites (11, 12, 18). Crucially, these agents normalize vascular permeability without the systemic pressor effects or proteinuria that are characteristic of VEGF-A blockade (7). This suggests that DRD2 activation facilitates vascular normalization rather than deprivation, thereby preserving the integrity of the vessel wall while quenching pathological signaling (11–13, 18).

### The dopaminergic challenge: a theranostic protocol

**Figure 1:**
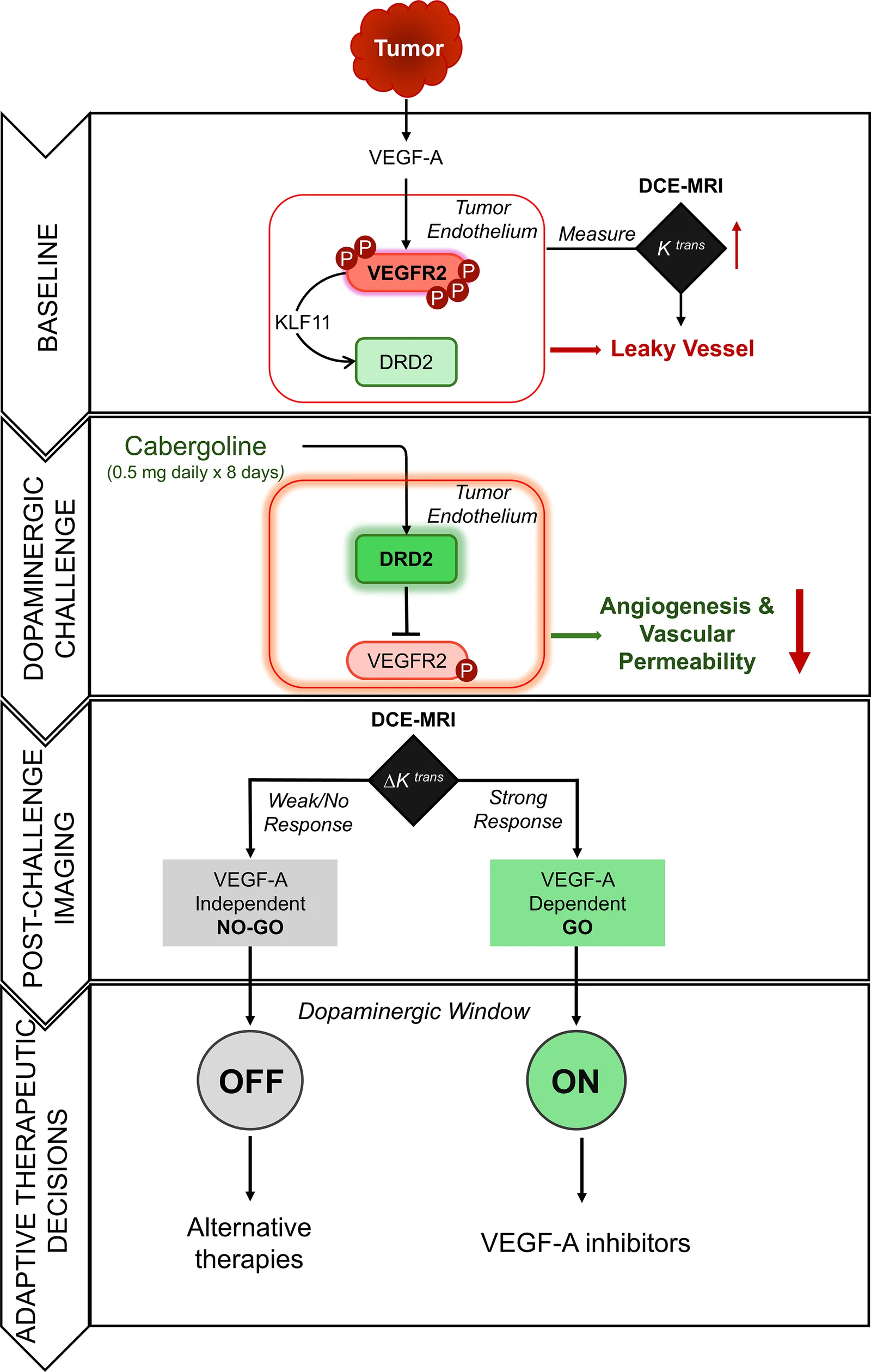
Schematic diagram showing the dopaminergic challenge workflow for VEGF-synchronized antiangiogenic therapy. Tumor-derived VEGF-A activates VEGFR2 phosphorylation in tumor endothelium, increasing vascular permeability, which can be quantified at baseline by Dynamic contrast-enhanced MRI (DCE-MRI) as elevated *K^trans^*. At the cellular level, VEGF-A signaling simultaneously induces endothelial DRD2 expression via KLF11 transcription factor, establishing a VEGF-dependent “dopaminergic window”. Brief administration of the FDA-approved DRD2 agonist (cabergoline) activates tumor endothelial DRD2, suppressing VEGFR2 phosphorylation and normalizing vascular permeability. Measurement of change in *K^trans^* through DCE-MRI pre- and post-challenge can functionally stratify tumors: a strong reduction indicates VEGF-A dependence, whereas weak or no response indicates VEGF-A independence. This DCE-MRI-guided readout directs the adaptive therapeutic decisions, supporting the use of VEGF-A inhibitors during active dopaminergic windows (ON state) or alternative therapies (OFF state). This theranostic protocol addresses antiangiogenic resistance by synchronizing therapy with real-time tumor vascular biology.

We propose exploiting this unique biology for a functional diagnostic protocol: the “Dopaminergic Challenge.” Because endothelial DRD2 expression is a proximal downstream consequence of VEGF-A activity (8), the acute vascular response to a DRD2 agonist serves as a live, physiological sensor of the tumor’s angiogenic drive. In this clinical workflow, a baseline assessment of vascular permeability is established via dynamic contrast-enhanced MRI to determine the volume transfer constant (*K^trans^*) (19). Following the oral administration of cabergoline using the dosing regimen that was previously shown to inhibit VEGF-A-induced vascular permeability in patients with OHSS (0.5 mg daily for 8 days) (20), the MRI will be performed again. A >40% reduction in the *K^trans^* would indicate a highly active, VEGF-A-dependent state, establishing a high probability of the existence of an “optimal window” for standard VEGF-A inhibitor administration (19). Conversely, the lack of a response would indicate that the tumor transitioned to VEGF-A-independent escape pathways, thereby rendering alternative therapeutic modalities more prudent and sparing the patient any adverse effects of continued (and futile) VEGF-A blockade therapy (**Figure 1**). Importantly, the ergot DRD2 agonist cabergoline-treated patients do not show cardiac valvular defects at durations and doses used to inhibit VEGF-A-induced angiogenesis and vascular permeability in OHSS and endometriosis (21).

### The translational roadmap

Validating this approach requires a multidimensional strategy that bridges molecular mapping with adaptive clinical trial design. Initial efforts must focus on high-resolution spatial transcriptomics and multiplex immunofluorescence to quantify the dopaminergic window (i.e., the DRD2-responsive VEGF-A window) and identify the precise histological tumor types and stages of progression where DRD2 expression is most prominent. Concurrently, preclinical studies are needed to confirm the correlation between DRD2-induced reductions in *K^trans^* and VEGF-A activity. A logical next step would be to initiate pilot “go/no-go” trials (22) wherein the response to the dopaminergic challenge dictates the administration of VEGF-A inhibitors. This approach will determine whether receptor-mediated feedback can accurately predict the therapeutic index of anti-angiogenic agents, ultimately transforming them from blunt instruments of deprivation into precision tools for vascular synchronization.

## Conclusion

Repurposing DRD2 agonists shifts the focus of anti-angiogenesis therapy from deprivation to synchronization. By engaging an endogenous feedback loop, we can sense the tumor’s requirements in real time and steer the vasculature toward a normalized state. This biologically resonant approach offers a pathway to anti-angiogenic therapy that is adaptive, selective, and functionally informed. However, clinical implementation of this theranostic strategy will require the prospective validation of DRD2 expression as a biomarker, standardization of dynamic contrast-enhanced-MRI protocols to ensure reproducible *K^trans^* measurements, and assessment of pharmacologic safety and feasibility in diverse oncological patient populations. The operational challenges of integrating functional imaging into adaptive trial designs and routine practice must also be addressed prior to broader adoption.

## Data Availability

The original contributions presented in the study are included in the article/supplementary material. Further inquiries can be directed to the corresponding author.
