# Correction: Beyond inhibition: harnessing the DRD2–VEGF-A feedback loop for precision anti-angiogenesis therapy in cancer

**DOI:** 10.3389/fonc.2026.1978685

**Published:** 2026-09-02

**Authors:** Manas Ranjan Sahu, Venu Akkanapally, Partha Sarathi Dasgupta, Sujit Basu

**Affiliations:** 1 Department of Pathology, Ohio State University, Columbus, OH, United States; 2 Chittaranjan National Cancer Institute, Kolkata, India; 3 Department of Internal Medicine, Division of Medical Oncology, Columbus, OH, United States; 4 Comprehensive Cancer Center, Ohio State University, Columbus, OH, United States

**Keywords:** angiogenesis, cancer, dopamine D2 receptor agonists, therapy, vascular endothelial growth factor receptor-2, vascular endothelial growth factor-A

In the published article, in the **Funding** statement, incorrect “NIH/NCI and DOD USA” grant information was provided. The “NIH and DOD USA” grants listed were not associated with this work. The corrected **Funding** statement appear below.

“The author(s) declared that financial support was received for this work and/or its publication. MR Sahu, V Akkanapally, and S Basu were supported by the Ohio State University Comprehensive Cancer Center fund to Sujit Basu. ”

The original version of this article has been updated.

